# Effects of simulated acid rain on rhizosphere microorganisms of invasive *Alternanthera philoxeroides* and native *Alternanthera sessilis*

**DOI:** 10.3389/fmicb.2022.993147

**Published:** 2022-09-09

**Authors:** Mengying He, Zexun Hua, Hanying Chen, Yao Liu, Yue Li, Zhen Zhang

**Affiliations:** ^1^College of Resources and Environment, Anhui Agricultural University, Hefei, Anhui, China; ^2^Institute of Botany, Chinese Academy of Sciences, Beijing, China

**Keywords:** simulated acid rain, invasive species, native species, rhizosphere microorganisms, microbial communities

## Abstract

Acid rain not only has serious harm to the environment, but also has the same threat to plants, but the invasive plant *Alternanthera philoxeroides* still grows well compared to the native plant *Alternanthera sessilis* under acid rain stress. However, the underlying mechanism of resistance to the acid rain environment in invasive *Alternanthera philoxeroides* remains unclear. In the current study, we comparatively analyzed the plant physiological characteristics, soil physicochemical properties, and rhizosphere microbial communities of invasive *A*. *philoxeroides* and native *A*. *sessilis* under different pH condition. The simulated acid rain had a significant inhibitory effect on the morphological and physiological traits of *A*. *philoxeroides* and *A*. *sessilis* and reduced the soil nutrient content. However, *A*. *philoxeroides* was more tolerant of acid rain. Compared with CK, simulated acid rain treatment at pH 2.5 significantly increased the Chao1, ACE, and Shannon indexes of *A*. *philoxeroides* microorganisms. Under simulated acid rain treatment at pH 2.5, the fungal flora Chao1, ACE and Shannon index were significantly higher than those of CK by 14.5%, 12.4%, and 30.4%, respectively. The dominant bacterial phyla of soil bacteria were Proteobacteria, Actinobacteria, Bacteroidota, Actinobacteria, Firmicutes, Myxococcota, Chloroflexi, Patescibacteria, Gemmatimonadota, Verrucomicrobiota, and Armatimonadota. The dominant fungi were Ascomycota, Basidiomycota, Rozellomycota, and Olpidiomycota. The bacterial and fungal diversity and structure of *A*. *philoxeroides* and *A*. *sessilis* showed the greatest difference between the pH 2.5 treatment and CK. Redundancy analysis showed that electrical conductivity (EC) and total phosphorus (TP) were the main factors changing the bacterial communities, and available phosphorus (AP), organic matter (OM), EC, and pH were the main factors changing the fungal communities. This study contributes to the microbial community structure of the invasive plant *A*. *philoxeroides* and provides a theoretical basis for studying the invasion mechanism of invasive plants under acid rain.

## Introduction

Acid rain has been a global environmental issue since 1980 and has had an adverse impact on human health and ecosystem functions (Liu et al., 2019; Shu et al., 2019). At present, China’s acid rain area accounts for about 40% of the land area (Lv et al., 2014), and it has become the third largest acid rain pollution area after Europe and North America (Ramlall et al., 2015). The impact of acid rain on terrestrial ecosystems has attracted extensive attention (Wei et al., 2020; Zhang et al., 2020). Plants are an important part of terrestrial ecosystems and are the direct and largest victims of acid rain pollution (Ramlall et al., 2015). When plants are exposed to acid rain stress, the wax on the surface of plant leaves is eroded, accumulating harmful cations, reducing the chlorophyll content and photosynthetic rate, resulting in leaf aging, affecting plant biomass, inhibiting plant growth, and causing death (Zhang et al., 2020). In addition, acid rain has significant effects on community structure and diversity of rhizosphere microorganisms (Shafer, 1992; Wang et al., 2014). At present, research on the effect of acid rain on plants mainly focuses on the damage mechanism of acid rain on crops and economic crops and the resistance mechanism in plants (Du et al., 2017; Zheng et al., 2022), while studies on the response mechanism of alien invasive plants to acid rain are relatively limited.

Biological invasion is a common environmental problem faced by countries all over the world, and is considered the second largest threat to biodiversity worldwide, forming a mono-dominant population by competing with local species, resulting in the reduction in local biodiversity (Wang et al., 2021a). *Alternanthera philoxeroides* is a malignant invasive weed native to South America. It is a perennial plant of the genus *Alternanthera* Forsk in Amaranthaceae. It propagates vegetatively by stem nodes (Pan et al., 2006). Because it can grow on land and water and has a strong reproductive ability and phenotypic plasticity, it has spread all over the Yangtze River region and southern provinces in China. It has had a huge impact on China’s ecosystems and social economy (Wang et al., 2021b). *Alternanthera sessilis* is a native species of the invasive plant *A*. *philoxeroides* in China. It occupies the same niche as *A*. *philoxeroides* and can coexist in the same plant communities (Sun et al., 2010). Field investigations have found that *A*. *philoxeroides* under acid rain stress showed a good growth trend (Guo et al., 2012), and comparison of the different performances of invasive species and related species facing environmental interference is an effective method to reveal species invasion (Wang et al., 2009). However, the underlying mechanism of resistance to acid rain in invasive *A*. *philoxeroides* remains unclear.

Soil is the largest recipient of acid rain in terrestrial ecosystem (Wei et al., 2017). Acid rain can change soil physicochemical properties and affect soil ecosystems (Wu et al., 2016b). Additionally, soil microorganisms are an important part of soil ecosystems (Falkowski et al., 2008), maintaining ecosystem stability and functional diversity by decomposing soil organic matter and participating in the circulation and flow of carbon, nitrogen, phosphorus, and sulfur in the ecosystem (Averill and Classen, 2014; Whitaker et al., 2014). When the ecosystem is disturbed by natural or human factors, microbial composition, structure, and function change accordingly (Chu et al., 2016). Most studies show that soil acidification caused by acid rain changes soil physicochemical properties (Mohsen and Elaheh, 2012; Liu et al., 2020), reduces soil nutrients, decreases soil microbial biomass, and adversely affects microbial community structure (Xu et al., 2015; Shu et al., 2021), thereby affecting plant growth and development (El-Tarabily et al., 2007). In some short-term experimental studies, the activity of soil microbial communities’ diversity and richness has a positive response to acid rain (Liu et al., 2017; Li et al., 2020). However, most of these studies are about the response of farmland and forest soil microbial community structure and function under acid rain conditions, while there is little information about the response of invasive plant soil microbial communities under acid rain conditions.

In the current study, we focused on the invasive plant *A*. *philoxeroides* and the native plant *A*. *sessilis*. We investigated the effects of acid rain at different pH values (2.5, 4.5, and 7.0) on the phenotypic characteristics, physiological, and ecological characteristics, soil physicochemical properties, and rhizosphere microbial communities of invasive *A*. *philoxeroides* and native *A*. *sessilis*. The ecological adaptability and tolerance of *A*. *philoxeroides* and *A*. *sessilis* under acid rain stress were analyzed to understand the invasion mechanism of *A*. *philoxeroides* when acid rain occurs and to provide theoretical support for research on invasive plants.

## Materials and methods

### Field experiment and soil sampling

The experiment was carried out in a greenhouse at Anhui Agricultural University, China (31°51′50′N, 117°14′48′E). Both *A*. *philoxeroides* and *A*. *sessilis* were collected from the terrestrial environment around Nangang (31°52′27′N, 116°43′34′E), Shushan District, Hefei City, Anhui Province, China. We selected *A*. *philoxeroides* and *A*. *sessilis* plants with good growth and similar stem diameters as cut seedlings and cut them short and evenly with one node (1.5 cm on both ends of the node). They were then cultivated in a warm room. When the seedlings grew to 2–4 cm, we selected seedlings with similar growth for the pot experiment.

According to acid rain monitoring in China, the simulated acid rain was configured into two acidic gradients with pH values of 2.5 and 4.5 according to the volume ratio of H_2_SO_4_:HNO_3_ (5:1; Liu et al., 2018). The seedlings of the two selected plants were transplanted into pots (19 cm × 13 cm × 5 cm) with 350 g nutrient soil. The contents of nitrogen, phosphorus, and potassium in soil were 11.5, 2.7, and 8.3 g/kg, respectively. Four seedlings were planted in each pot, and six replicates were set for each treatment. The experiment was carried out in a greenhouse (EAV = 500 lux, illumination for 12 h, Rh = 75%) in September 2020. Acid rain or deionized water (control, CK) was sprayed on the plants every 2 days. Each application was carried out using a 2-L sprayer 20 cm above the plant top leaves, with 200 ml of each solution (calculated by annual precipitation in Hefei). During the experiment, the pot positions were randomly changed every day to reduce the effects of environmental differences, such as light and temperature, on plant growth. The experiment lasted 40 days.

### Measurement of the morphological and physiological indexes of *Alternanthera philoxeroides* and *Alternanthera sessilis*

After the experiment, plant height was measured using a ruler. The plant was placed in an envelope and dried in an oven at 65°C until a constant weight. The plant dry weight was measured using an electronic balance with an accuracy of 0.001 g. The chlorophyll content in plant leaves was determined using the acetone method (Dolatabadian et al., 2013). The photosynthesis index (net photosynthetic rate—Pn; stomatal conductance—Gs; intercellular carbon dioxide concentration—Ci; and transpiration rate—Tr) was measured using a portable photosynthesis system (6400XT, LI-COR, Lincoln, NE, United States).

### Soil physicochemical characteristics

The soil pH was determined using the potentiometric method, and the electrical conductivity (EC) was measured using a conductivity meter (Meng et al., 2019). The organic matter (OM) was determined using the external heating potassium dichromate volumetric method(Wu et al., 2022). Total nitrogen (TN) was determined using the semi-micro Kjeldahl method, and total phosphorus (TP) and total potassium (TK) were measured using the NaOH melting method. Ammonium nitrogen (AN) was determined using Indigo colorimetry. The Olsen method was used to determine the available phosphorus (AP) content (Ren et al., 2021).

### Genomic DNA extraction, PCR amplification, and Miseq sequencing

At the end of the experiment, fresh rhizosphere soil of two species under pH 2.5, pH 4.5 and CK was collected by root shaking method in sterile centrifuge tubes and recorded as AP1, AP2, AP3, AS1, AS2, and AS3, respectively. Genomic DNA was extracted from soil samples using an OMEGA soil DNA extraction kit (MP Biomedicals, United States). The DNA extract was examined on 1% agarose gel, and NanoDrop 2000 (Thermo Scientific, Wilmington, NC, United States) was used to determine the DNA concentration and purity. The V3–V4 region of the bacterial 16 s RNA gene using primers (338F: 5′-ACTCCTACGGGAGGCAGCAG-3′; 806R: 5′-GGACTACHVGGGTWTCTAAT-3′) was amplified by ABI GeneAmp® 9,700(ABI, CA, United States). The fungal ITS region was amplified using the following primers (ITS1F:5′-CTTGGTCATTTAGAGGAAGTAA-3′; ITS2R:5′-GCTGCGTTCTTCATCGATGC-3′). The PCR amplification of 16 s RNA gene was performed as follows: initial denaturation at 95°C for 3 min, followed by 27 cycles of denaturing at 95°C for 30 s, annealing at 55°C for 30 s and extension at 72°C for 45 s, and single extension at 72°C for 10 min, and end at 10°C. Similarly, the PCR amplification of fungal ITS region was only 35 cycles, and its parameters were consistent with the PCR amplification program of 16 s RNA. The PCR reaction mixture contained Takara rTaq DNA polymerase in a 20 μl reaction system with 2 μl of 10× Fastpfu buffer, 2 μl of 2.5 mM dNTPs, 0.8 μl forward primer (5 μM), 0.8 μl reverse primer (5 μM), 0.2 μl rTaq polymer, 0.2 μl BSA, 10 ng template DNA, and supplemented with ddH_2_O to 20 μl. Each sample was replicated three times. The PCR products of the same sample were mixed and detected using 2% agarose gel electrophoresis. The PCR product was recovered using the AxyPrepDNA gel Recovery Kit (AXYGEN company), and the PCR product was detected and quantified using the QuantiFluor™—ST blue fluorescence quantitative system (Promega, United States). Sequencing was carried out on the Illumina MiSeq PE300 platform.

The sequencing file was transferred from FASTP0.19.6 software filtering (Chen et al., 2018) to FLASH1.2.7 (Mago and Salzberg, 2011) to screen and merge the sequences, using the following steps: (1) The bases were filtered with a mass value of less than 20 at the tail of reads. A 50 bp window was set; if the average mass value in the window was less than 20, the back-end bases were cut from the window. The reads were filtered with a mass value of less than 50 bp after quality control, and reads containing N bases were removed. (2) According to the overlapping relationship between PE reads, paired reads were spliced into a sequence, and the minimum overlap length was 10 bp. (3) The maximum allowable mismatch ratio of the overlapping regions of splicing sequences was 0.2, and the non-conforming sequences were screened. (4) The samples were distinguished according to the barcodes and primers at the beginning and end of the sequences, and the sequence direction was adjusted.

### Statistical analysis

UPARSE (version 7.0.1090) was used for operational taxonomic unit (OTU) clustering of non-repeat sequences (excluding single sequences) according to a 97% similarity to identify and delete chimeric sequences (Stakenrandt and Goebel, 1994; Edgar, 2013). The ɑ-diversity (Shannon, Simpson, CHAO1, ACE) of the microbial communities was calculated using QIIME1.9.1 software. The β-diversity and difference OTUs were calculated by the vegan and edgeR packages in R language (Version 3.5.5) software. The correlation analysis between microbial diversity, relative abundance, and soil physicochemical properties was performed using Pearson and Spearman correlations. All experimental data were analyzed using Excel and IBM SPSS Statistics.

## Results

### Effects of simulated acid rain on the morphological and physiological indexes of *Alternanthera philoxeroides* and *Alternanthera sessilis*

**Figure 1:**
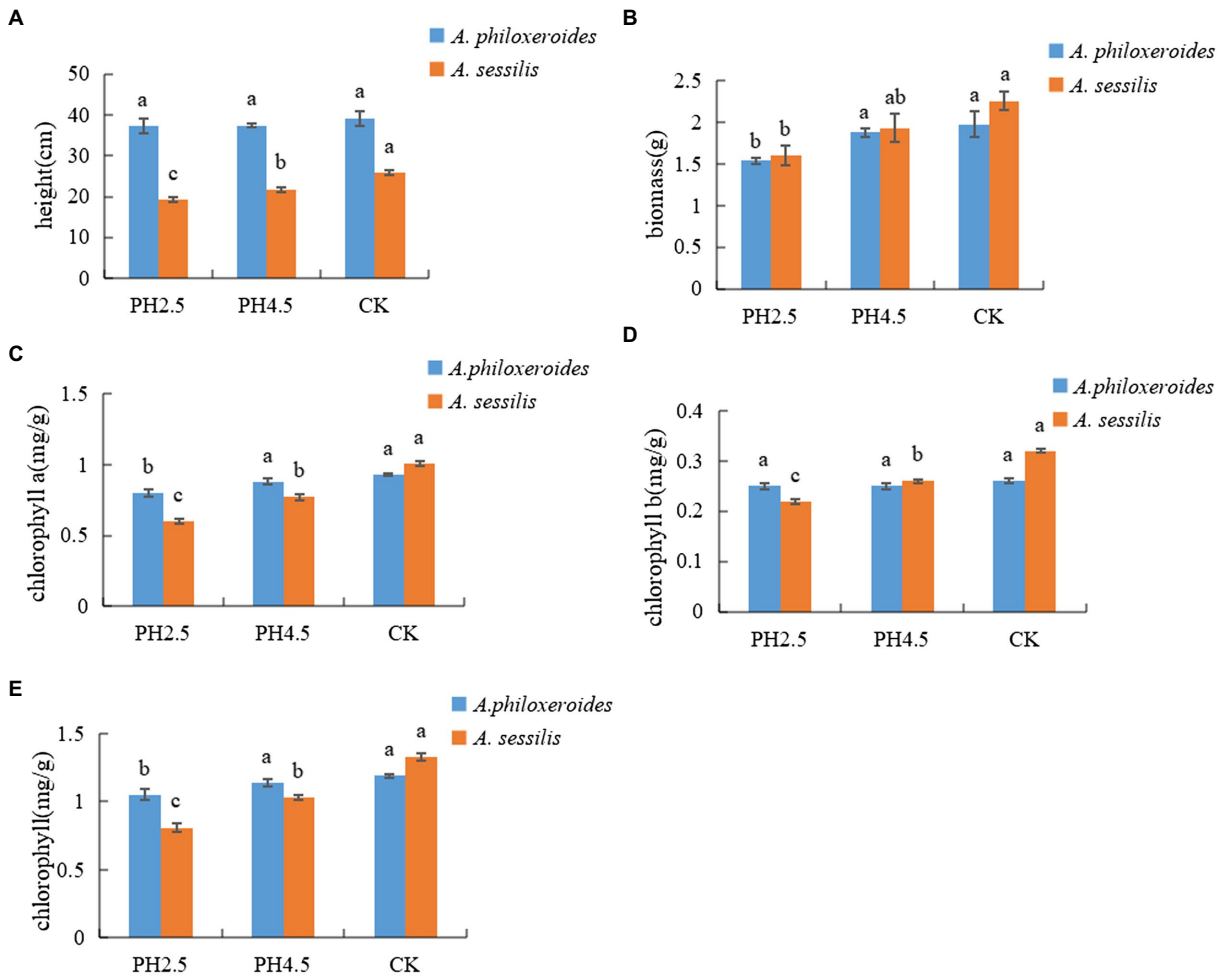
Effects of simulated acid rain on morphological and physiological indexes(**A**—height; **B**—biomass; **C**—chlorophyll a; **D**—chlorophyll b; **E**—chlorophyll.) of *Alternanthera philoxeroides* and *Alternanthera sessilis*. Different letters in the same species indicate significant differences among the different treatments (*p* < 0.05).

The plant height and biomass of *A*. *philoxeroides* and *A*. *sessilis* decreased with a decrease in pH value and reached a minimum at pH 2.5. Compared with CK, plant height of *A*. *philoxeroides* and *A*. *sessilis* decreased by 4.8% and 25.5%, and biomass of *A*. *philoxeroides* and *A*. *sessilis* decreased by 22.2% and 29.2%, respectively (Figure 1). With a decrease in pH value, the contents of chlorophyll a, chlorophyll b, and total chlorophyll decreased. Compared with CK, chlorophyll of *A*. *philoxeroides* and *A*. *sessilis* decreased by 14.0% and 40.6%, chlorophyll b of *A*. *philoxeroides* and *A*. *sessilis* decreased by 3.8% and 31.3%, and total chlorophyll of *A*. *philoxeroides* and *A*. *sessilis* decreased by 11.8% and 39.1%, respectively. Only the intercellular carbon dioxide concentration of *A*. *philoxeroides* increased and then decreased with the decrease in pH value, and its other parameters and all gas exchange parameters of *A*. *sessilis* showed a decreasing trend. Compared with CK, the net photosynthetic rate (Pn) decreased by 44.3% and 54.1%, the stomatal conductance (Gs) decreased by 60.5% and 60.7%, the intercellular carbon dioxide concentration (Ci) decreased by 7.0% and 3.4%, and the transpiration rate (Tr) decreased by 47.9% and 59.6%, respectively (Table 1).

**Table 1:** Effects of simulated acid rain on plant gas exchange parameters of *Alternanthera philoxeroides* and *Alternanthera sessilis.*

Species	Treatment	Pn (mmol·m^−2^·s^−1^)	Gs (mol·m^−2^·s^−1^)	Ci (mmol·m^−2^·s^−1^)	Tr (mmol·m^−2^·s^−1^)
*Alternanthera philoxeroides*	pH 2.5	6.23 ± 0.22c	0.075 ± 0.006c	212.78 ± 4.74a	2.19 ± 0.15c
	pH 4.5	8.57 ± 0.21b	0.118 ± 0.009b	233.07 ± 9.72a	2.86 ± 0.13b
	CK	11.19 ± 0.59a	0.190 ± 0.011a	228.71 ± 4.94a	4.20 ± 0.13a
*Alternanthera sessilis*	pH 2.5	2.08 ± 0.13c	0.048 ± 0.004c	251.09 ± 5.66a	1.15 ± 0.10b
	pH 4.5	2.78 ± 0.16b	0.062 ± 0.003b	255.15 ± 3.31a	1.48 ± 0.10b
	CK	4.53 ± 0.28a	0.122 ± 0.007a	259.85 ± 4.23a	2.85 ± 0.16a

Pn, net photosynthetic rate; Gs, stomatal conductance; Ci, intercellular carbon dioxide concentration; Tr, transpiration rate. Data are the average values ± standard error. Data are the average values ± standard error. Different letters in the same row indicate significant differences among the different treatments (*p* < 0.05).

### Effects of simulated acid rain on soil physicochemical properties

The soil pH of *A*. *philoxeroides* decreased with the decrease in pH value of simulated acid rain (Table 2). The original soil pH value under CK was 6.93, while the soil pH value under the simulated acid rain treatment (pH 2.5) was 6.30, resulting in a decrease of 9.1%. The change trend of organic matter was the same as that of soil pH, reaching the lowest at pH 2.5, which was 7.6% lower than that of CK. The electrical conductivity showed an increasing trend (Table 2) and was 188.2% higher under the simulated acid rain treatment (pH 2.5) than under CK (*p* < 0.05). The total nitrogen and available phosphorus changed with the increase in pH value of simulated acid rain (Table 2), and were 2.5% and 21.0% higher, respectively, under the simulated acid rain treatment at pH 4.5, and 2.5% and 10.4% lower, respectively, under the simulated acid rain at pH 2.5 than under CK (*p* < 0.05). Total phosphorus, total potassium, and ammonium nitrogen in *A*. *philoxeroides* soil were not significantly affected by acid rain (Tables 2; *p* > 0.05).

**Table 2:** Effects of simulated acid rain on soil physicochemical properties of *Alternanthera philoxeroides* and *Alternanthera sessilis.*

Species	*A*. *philoxeroides*			*A*. *sessilis*		
Treatment	pH 2.5	pH 4.5	CK	pH 2.5	pH 4.5	CK
pH	6.30 ± 0.02b	6.79 ± 0.09a	6.93 ± 0.06a	6.05 ± 0.09b	6.57 ± 0.12a	6.82 ± 0.05a
EC μs/cm	597.4 ± 66.2a	261.4 ± 34.4b	207.3 ± 14.7b	602.4 ± 67.0a	305.8 ± 23.9b	289.4 ± 9.4b
OM g/kg	647.9 ± 4.3b	689.7 ± 12.0a	701.3 ± 5.9a	633.9 ± 12.0b	710.6 ± 16.2a	724.5 ± 5.9a
TN g/kg	11.5 ± 0.05c	12.1 ± 0.05a	11.8 ± 0.07b	11.7 ± 0.14b	12.3 ± 0.18a	11.9 ± 0.09ab
TP g/kg	2.7 ± 0.06a	2.9 ± 0.04a	2.9 ± 0.13a	2.4 ± 0.11b	2.8 ± 0.19ab	3.3 ± 0.11a
TK g/kg	7.5 ± 0.5a	7.8 ± 0.3a	8.3 ± 0.3a	8.1 ± 0.2b	8.7 ± 0.1ab	9.2 ± 0.2a
AN mg/kg	28.0 ± 0.8b	33.8 ± 1.8a	31.3 ± 0.5ab	23.4 ± 0.8c	28.7 ± 0.6b	31.7 ± 0.8a
AP mg/kg	75.5 ± 1.4c	102.0 ± 3.8a	84.3 ± 2.6b	77.0 ± 2.1b	108.5 ± 1.3a	101.0 ± 4.8a

EC, electrical conductivity; OM, organic matter; TN, total nitrogen; TP, total phosphorus; TK, total potassium; AN, ammonium nitrogen; AP, available phosphorus. Data are the average values ± standard error. Different letters in the same row indicate significant differences among the different treatments (*p* < 0.05).

In *A*. *sessilis*, the soil pH, organic matter, total phosphorus, total potassium, available phosphorus, and ammonium nitrogen showed a downward trend with a decrease in the pH of acid rain (Table 2). Under the simulated acid rain treatment at pH 2.5, these parameters were significantly lower than CK (*p* < 0.05), and the maximum decreases were 11.3%, 12.5%, 27.8%, 12.0%, 26.2%, and 29.7%, respectively. The electrical conductivity showed an increasing trend (Table 2) and was 108.2% higher under the simulated acid rain treatment at pH 2.5 than under CK (*p* < 0.05). There was no significant difference in soil total nitrogen between the acid rain treatments and CK (Tables 2; *p* > 0.05). Acid rain reduces the content of soil nutrients. Under the same treatment (pH 2.5 and pH 4.5), the decline in soil pH, OM, TP, TK, AN, and AP under *A*. *philoxeroides* was less than that of *A*. *sessilis*, and the influence of *A*. *philoxeroides* soil nutrients under acid rain was less than that of *A*. *sessilis*.

### Effects of simulated acid rain on microbial diversity

With the decrease in simulated acid rain pH, the bacterial flora richness index (Chao1 and ACE) and Shannon index of *A*. *philoxeroides* increased and were 6.8%, 8.8%, and 5.5% higher, respectively, under simulated acid rain at pH 2.5 than under CK (*p* < 0.05). The Simpson index showed a downward trend and was 30.2% lower under the simulated acid rain treatment at pH 2.5 than under CK (*p* < 0.05). The richness index of fungal flora changed with the increase in pH value of simulated acid rain, and was significantly higher under the simulated acid rain at pH 2.5 than under CK (18.0% and 17.0%; *p* < 0.05). The Shannon index showed an increasing trend, which was significantly higher than under the simulated acid rain at pH 2.5 than under CK (14.8%; *p* < 0.05). The Simpson index showed a downward trend and was 27.9% lower under the simulated acid rain at pH 2.5 than under CK (*p* < 0.05).

The bacterial flora richness index and diversity index (Simpson and Shannon) of *A*. *sessilis* were not significantly affected by acid rain (*p* > 0.05). The fungal flora richness index (Chao1 and ACE) and Shannon index changed with the increase in pH value of simulated acid rain. Under the simulated acid rain treatment at pH 2.5, they were significantly higher than those of CK by 14.5%, 12.4%, and 30.4%, respectively (*p* < 0.05). The Simpson index increased first and then decreased, and was significantly lower under simulated acid rain at pH 2.5 than under CK (37.3%; *p* < 0.05). Under acid rain stress, the change in the bacterial richness and diversity of *A*. *philoxeroides* was greater than that of *A*. *sessilis*. The change in fungal richness was greater than that of *A*. *sessilis*, and the change in diversity was less than that of *A*. *sessilis* (Table 3).

**Table 3:** Effects of simulated acid rain on microbial diversity.

Species	Microbial communities	Treatment	Flora richness index		Flora diversity index	
			Chao1	ACE	Shannon	Simpson
*Alternanthera philoxeroides*	Bacteria	pH 2.5	1642.2 ± 45.3a	1644.5 ± 38.6a	5.8 ± 0.06a	0.0064 ± 0.0004b
		pH 4.5	1537.9 ± 25.0b	1533.2 ± 21.6b	5.6 ± 0.04b	0.0079 ± 0.0006a
		CK	1530.3 ± 98.0b	1500.3 ± 59.4b	5.5 ± 0.10c	0.0096 ± 0.0023a
	Fungus	pH 2.5	334.5 ± 32.7a	328.8 ± 24.5a	3.1 ± 0.06a	0.093 ± 0.008b
		pH 4.5	261.9 ± 28.9b	260.1 ± 29.5b	2.8 ± 0.05b	0.128 ± 0.003a
		CK	283.5 ± 14.35b	281.0 ± 12.8b	2.7 ± 0.06b	0.129 ± 0.008a
*Alternanthera sessilis*	Bacteria	pH 2.5	1417.1 ± 75.8a	1398.7 ± 59.77a	5.5 ± 0.08a	0.0087 ± 0.0009a
		pH 4.5	1413.4 ± 56.7a	1398.4 ± 51.6a	5.5 ± 0.03a	0.0088 ± 0.0003a
		CK	1395.5 ± 47.4a	1389.6 ± 40.1a	5.6 ± 0.06a	0.0085 ± 0.0011a
	Fungus	pH 2.5	252.0 ± 12.2a	248.7 ± 11.6a	3.0 ± 0.28a	0.106 ± 0.03c
		pH 4.5	216.2 ± 19.5b	218.0 ± 20.1b	2.2 ± 0.06c	0.209 ± 0.02a
		CK	220.0 ± 19.6b	221.2 ± 20.4b	2.3 ± 0.11b	0.169 ± 0.01b

Data are the average values ± standard error. Different letters in the same row indicate significant differences among the different treatments (*p* < 0.05).

### Principal component analysis of simulated acid rain on microbial community composition

**Figure 2:**
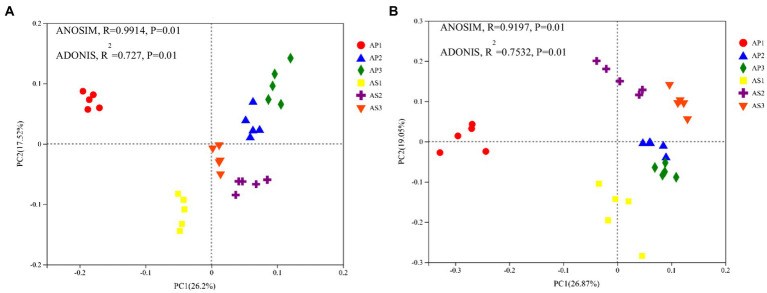
Effects of simulated acid rain on the community structure of soil bacteria **(A)** and fungi **(B)**. AP1, AP2, and AP3 indicate that *Alternanthera philoxeroides* was treated with simulated acid rain at pH 2.5, 4.5, and 7.0, and AS1, AS2, and AS3 indicate that *A*. *sessilis* was treated with simulated acid rain at 2.5, 4.5, and 7.0.

To intuitively reflect the differences in microbial communities’ composition and structure between *A*. *philoxeroides* and *A*. *sessilis* under different acid rain treatments, principal coordinate analysis was carried out using the Bray–Curtis distance algorithm (Figure 2). The cumulative contribution rate of the first two principal components (PC1 and PC2) was 43.72% (Figure 2A), indicating that these two principal components were the main contributors to the difference in bacterial ommunity composition between *A*. *philoxeroides* and *A*. *sessilis* under simulated acid rain. Under each treatment, the soil bacteria of *A*. *philoxeroides* were clustered in the positive semi-axis of PC2, and the soil bacteria of *A*. *sessilis* were clustered in the negative semi-axis of PC2. Furthermore, the soil bacteria of *A*. *philoxeroides* and *A*. *sessilis* were all in the negative semi-axis of PC1 under pH 2.5 treatment. The ANOSIM and ADONIS methods were used to analyze the similarity of soil fungal communities under different acid rain treatments. Simulated acid rain changed the bacterial community structures of *A*. *philoxeroides* and *A*. *sessilis*. The cumulative contribution rate of the first two principal components to the difference in the composition and structure of soil fungal communities between *A*. *philoxeroides* and *A*. *sessilis* was 45.92% (Figure 2B). Under the pH 2.5 treatment, the soil fungi of *A*. *philoxeroides* aggregated in the negative semi-axis of PC1, while the soil fungi of *A*. *philoxeroides* aggregated in the positive semi-axis of PC1 under the pH 4.5 treatment and CK. Under pH 2.5 treatment, the soil fungi of *A*. *sessilis* aggregated in the negative semi-axis of PC2, while the soil fungi of *A*. *sessilis* aggregated in the positive semi-axis of PC2 under the pH 4.5 treatment and CK. Correlation analysis showed that simulated acid rain changed the fungal community structure of *A*. *philoxeroides* and *A*. *sessilis*.

### Effects of simulated acid rain on microbial community composition

**Figure 3:**
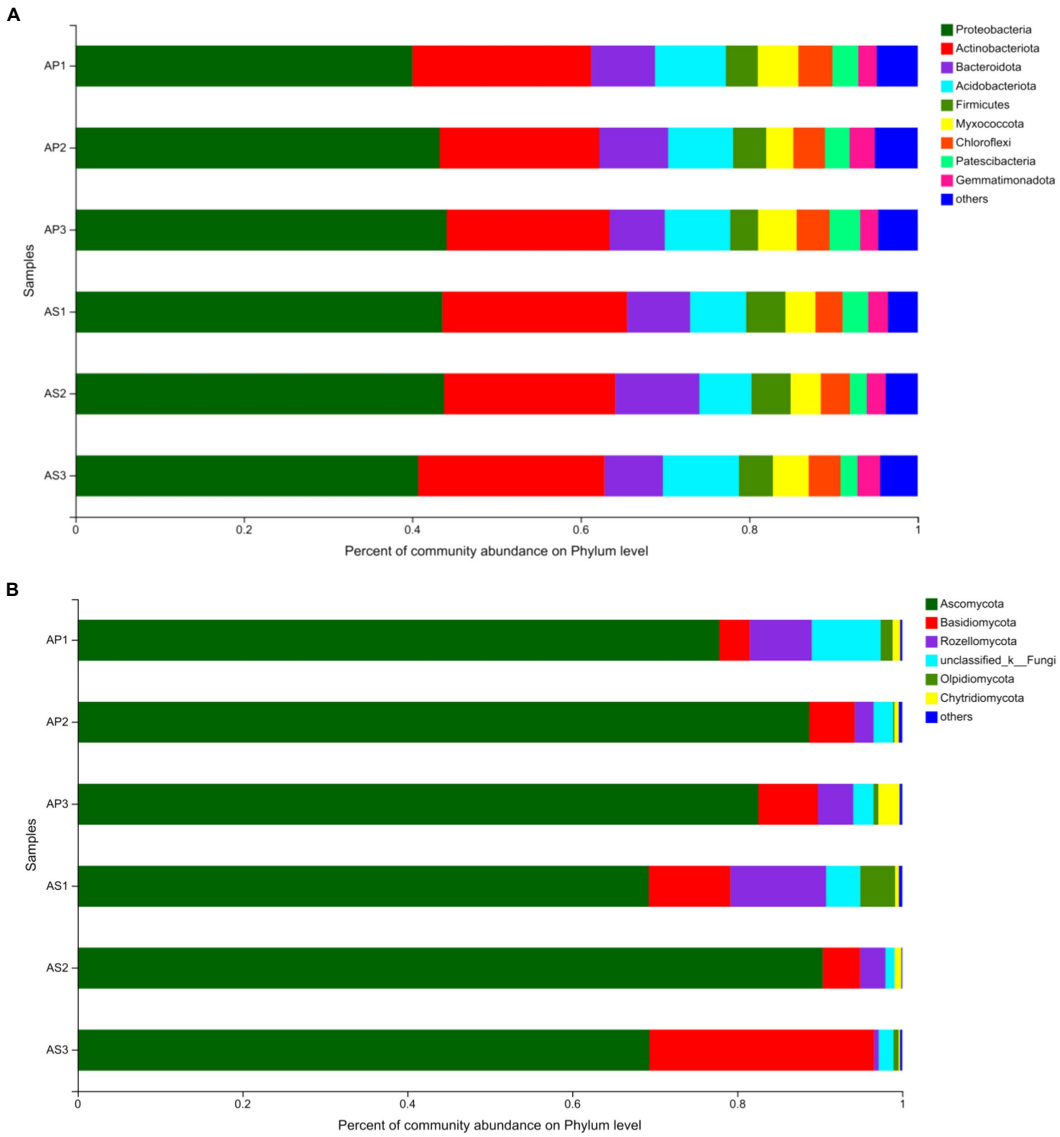
Effects of simulated acid rain on the composition and relative abundance of soil bacterial **(A)** and fungal **(B)** communities.

**Figure 4:**
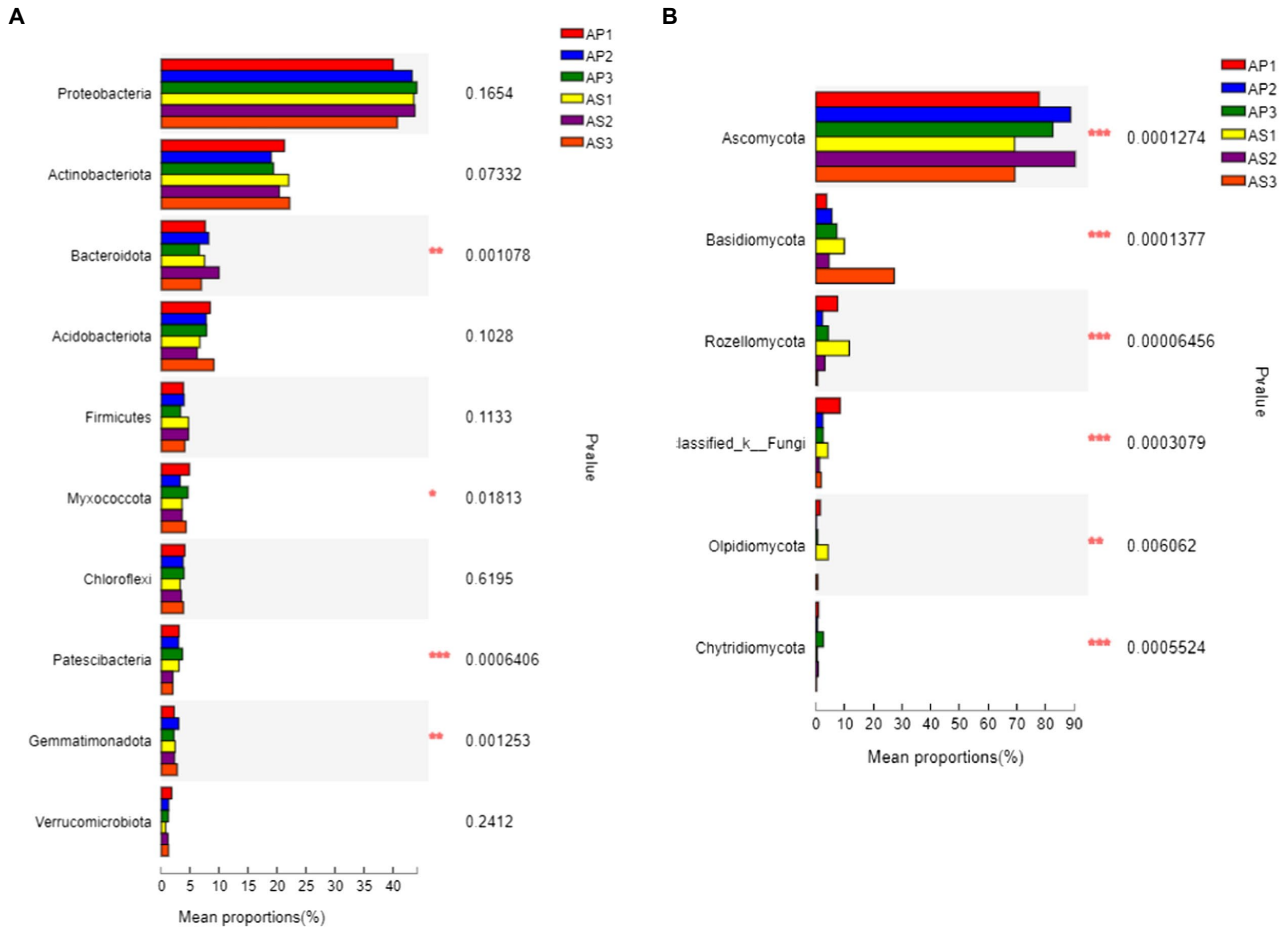
Difference analysis of dominant bacteria groups at the level of soil bacterial **(A)** and fungal **(B)** communities under simulated acid rain. ^*^p < 0.05, ^**^p < 0.01, ^***^p < 0.005.

Under simulated acid rain treatment, the dominant bacteria (relative abundance > 1%) in the soil shared by *A*. *philoxeroides* and *A*. *sessilis* were Proteobacteria, Actinobacteriota, Bacteroidota, Acidobacteriota, Firmicutes, Myxococcota, Chloroflexi, Patescibacteria, Gemmatimonadota, Verrucomicrobiota, and Armtimonadota (Figure 3A). Among them, the proportion of Proteobacteria, Actinobacteriota, Bacteroidota, Acidobacteriota, and Firmicutes was as high as 80%. Under CK, there was no significant difference in the relative abundance of bacterial communities between *A*. *philoxeroides* and *A*. *sessilis* in the five phyla (Figure 4A, *P* > 0.05). With the simulated acid rain treatment, the relative abundance of Bacteroidota in *A*. *sessilis* soil was significantly higher than that in the other treatments only under the treatment at pH 4.5 (*p* < 0.05). Therefore, different degrees of simulated acid rain had little effect on the composition of bacterial communities at the soil phyla level in *A*. *philoxeroides* and *A*. *sessilis*.

The dominant fungi in the *A*. *philoxeroides* and *A*. *sessilis* soil were Ascomycota, Basidiomycota, Rozellomycota, and Olpidiomycota (Figure 3B). There were significant differences in the relative abundance of the dominant fungal flora of *A*. *philoxeroides* and *A*. *sessilis* under different simulated acid rain treatments (Figure 4B). With the simulated acid rain treatment, the relative abundance of Ascomycota in both plants increased first and then decreased. Under the pH 2.5 treatment, the relative abundance of Ascomycota in *A*. *philoxeroides* was significantly higher than that in *A*. *sessilis* (12.3%, *p* < 0.05), and the relative abundance of Basidiomycota was significantly inhibited by acid rain (*p* < 0.05). The relative abundance of Basidiomycota in *A*. *philoxeroides* decreased by 23.8%–49.0%, and that in *A*. *sessilis* decreased by 63.6%–83.3%. The relative abundance of Rozellomycota in *A*. *philoxeroides* under the pH 2.5 treatment was 1.8 times higher than that of CK. The relative abundance of Rozellomycota in *A*. *sessilis* under acid rain treatment (pH2.5 and pH4.5) was 20.4 times and 5.4 times higher, respectively, than that of CK.

### Functional prediction of microbial communities under simulated acid rain treatments

**Figure 5:**
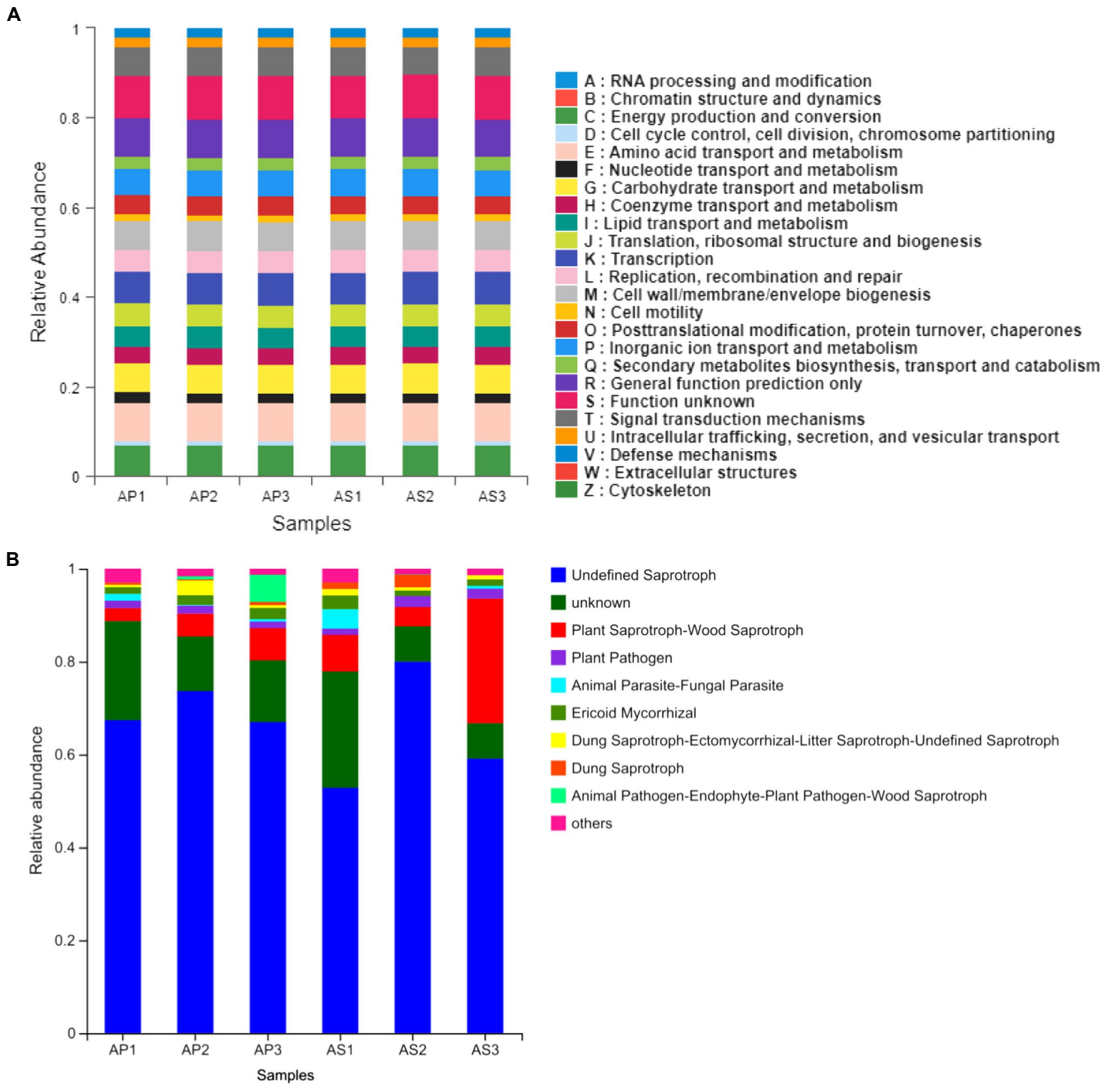
Function prediction of simulated acid rain on soil bacteria **(A)** and fungi **(B)** of *Alternanthera philoxeroides* and *Alternanthera sessilis*.

Through the functional prediction of the soil microbial communities of *A*. *philoxeroides* and *A*. *sessilis* under simulated acid rain treatment, the functional prediction of the soil bacteria of *A*. *philoxeroides* and *A*. *sessilis* under acid rain treatment was relatively similar (Figure 5A). Those with relatively large functional abundance were as follows: S (Function unknown), E (Amino acid transport and metabolism), R (General function prediction only), K (Transcription), C (Energy production and conversion), M (Cell wall/membrane/envelope biogenesis), T (Signal transduction mechanisms), G (Carbohydrate transport and metabolism), and P (Inorganic ion transport and metabolism). Acid rain did not change the function of the soil bacteria of *A*. *philoxeroides* and *A*. *sessilis*.

The soil fungal function of *A*. *philoxeroides* and *A*. *sessilis* changed under acid rain treatment (Figure 5B). With the decrease in pH of simulated acid rain, the abundance of undefined saprotrophs and plant pathogens of *A*. *philoxeroides* increased first and then decreased, but both were higher than that in CK, with maximum increases of 10.0% and 20.7%, respectively. The abundance of plant saprotroph–wood saprotroph and ericoid mycorrhizal decreased, with maximum decreases of 59.4% and 39.5%, respectively. The abundance of undefined saprotrophs and plant pathogens of *A*. *sessilis* increased first and then decreased, with decreases of 10.6% and 36.6%, respectively. The relative abundance of plant pathogens of *A*. *sessilis* was highest under the simulated acid rain treatment at pH 4.5. Plant saprotroph–wood saprotroph decreased first and then increased, with a maximum decrease of 84.5%. Ericoid mycorrhizal decreased first and then increased, with an increase of 113.8% and a decrease of 14.9%.

### Response of microbial communities and diversity to environmental factors under simulated acid rain treatments

Correlation analysis between environmental factors and microbial diversity showed that (Table 4) bacterial Chao1 and ACE indexes responded significantly to soil total nitrogen, total potassium, and available phosphorus (*p* < 0.05), and the bacterial Shannon index responded significantly to total potassium (*p* < 0.05). The fungal Chao1 index responded significantly to electrical conductivity, total nitrogen, total potassium, organic matter, and available phosphorus (*p* < 0.05). The fungal ACE index responded significantly to total nitrogen, total potassium, organic matter, and available phosphorus (*p* < 0.05). The fungal Shannon index responded significantly to pH, electrical conductivity, total nitrogen, total phosphorus, total potassium, organic matter, and available phosphorus (*p* < 0.05). The Simpson index responded significantly to pH, electrical conductivity, total nitrogen, total potassium, organic matter, and available phosphorus (*p* < 0.05).

**Table 4:** Correlation analysis between soil microbial diversity and physicochemical properties.

	Microbial diversity	pH	EC	TN	TP	TK	OM	AN	AP
Bacteria	Chao1	0.060	0.141	−0.471^*^	−0.079	−0.653^*^	−0.256	0.115	−0.420^*^
	ACE	0.028	0.185	−0.453^*^	−0.111	−0.634^*^	−0.274	0.111	−0.404^*^
	Shannon	−0.091	0.310	−0.196	0.040	−0.385^*^	−0.275	0.039	−0.305
	Simpson	0.192	−0.343	0.093	0.019	0.288	0.329	0.127	0.273
Fungus	Chao1	−0.196	0.403^*^	−0.561^*^	−0.193	−0.603^*^	−0.464^*^	−0.035	−0.611^*^
	ACE	−0.170	0.357	−0.562^*^	−0.159	−0.566^*^	−0.437^*^	−0.023	−0.595^*^
	Shannon	−0.471^*^	0.607^*^	−0.584^*^	−0.422^*^	−0.499^*^	−0.775^*^	−0.271	−0.785^*^
	Simpson	0.396^*^	−0.522^*^	0.564^*^	0.312	0.452^*^	0.711^*^	0.236	0.787^*^

* indicates *p* < 0.05.

**Figure 6:**
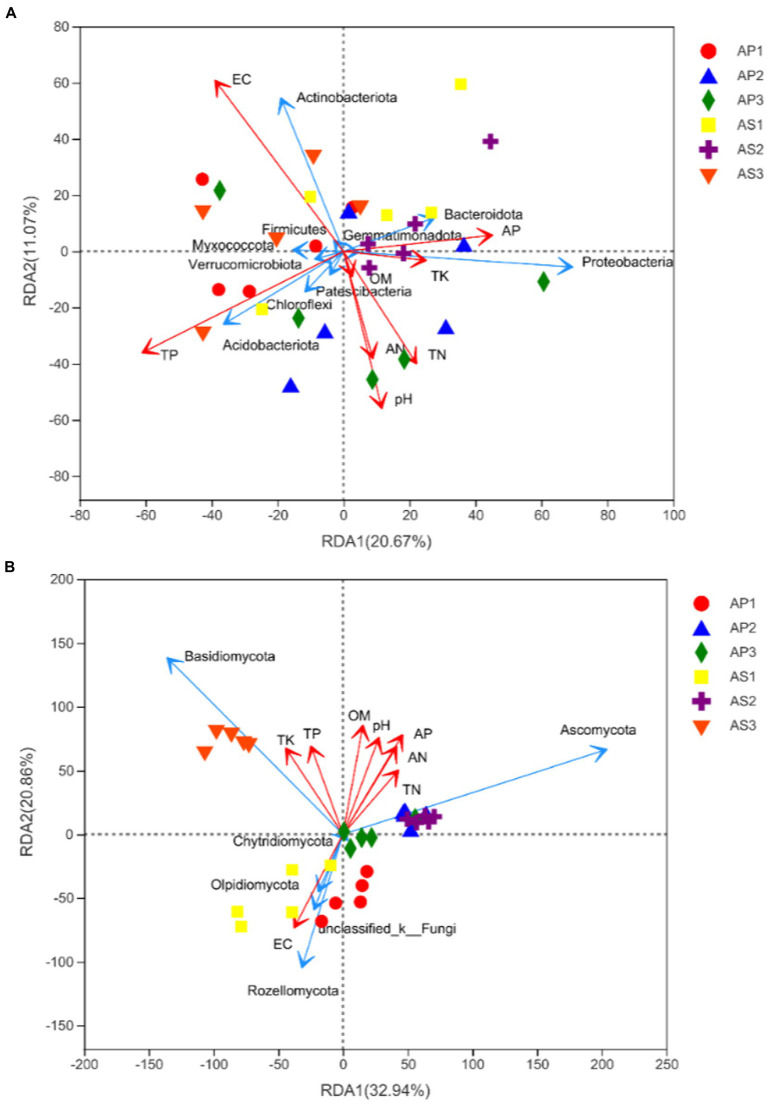
Redundancy analysis of the response of soil bacterial **(A)** and fungal **(B)** communities to environmental factors.

Bacterial RDA analysis showed that the cumulative explained variation of the first axis and the second axis was 31.74% (Figure 6A), and EC and TP were the main factors that changed bacterial communities. Correlation analysis showed that (Table 5) soil EC was significantly positively correlated with Actinobacteriota (*p* < 0.05), and TP was significantly positively correlated with Acidobacteriota (*p* < 0.05). Fungal RDA showed that the cumulative explanatory variation of the first axis and the second axis was 53.8% (Figure 6B), in which AP, OM, EC, and pH were the main factors that changed fungal communities. Correlation analysis showed that AP was significantly positively correlated with Ascomycota (*p* < 0.05) and significantly negatively correlated with Rozellomycota, Olpidiomycota, and Chytridiomycota (*p* < 0.05). OM was significantly negatively correlated with Rozellomycota and Olpidiomycota (*p* < 0.05), and EC showed a significant positive correlation with Rozellomycota and Olpidiomycota (*p* < 0.05). There was a significant negative correlation between pH and Rozellomycota and Olpidiomycota (*p* < 0.05).

**Table 5:** Correlation analysis between soil microbiota level and physicochemical properties.

Microorganism		pH	EC	TN	TP	TK	OM	AN	AP
Bacteria	Proteobacteria	0.118	−0.232	−0.006	−0.333	0.073	0.031	−0.049	0.236
	Actinobacteriota	−0.312	0.373^*^	−0.360	−0.149	0.042	−0.017	−0.173	−0.107
	Bacteroidota	−0.225	0.111	0.348	−0.303	−0.054	0.070	−0.088	0.419^*^
	Acidobacteriota	0.217	−0.145	0.014	0.500^*^	0.004	0.083	0.220	−0.181
	Firmicutes	−0.285	0.344	0.377^*^	0.035	0.249	−0.092	−0.383^*^	−0.008
	Myxococcota	−0.090	0.036	−0.417^*^	0.098	−0.054	−0.083	0.031	−0.311
	Chloroflexi	0.064	−0.081	0.016	0.273	−0.088	−0.074	0.034	−0.225
	Patescibacteria	0.037	0.085	−0.335	−0.263	−0.395^*^	−0.394^*^	−0.148	−0.550^*^
	Gemmatimonadota	0.214	−0.235	0.234	0.283	0.172	0.247	0.362^*^	0.434^*^
	Verrucomicrobiota	0.180	0.001	0.205	0.235	−0.040	0.133	0.130	−0.042
Fungus	Ascomycota	0.327	−0.357	0.378^*^	0.053	−0.113	0.296	0.252	0.516^*^
	Basidiomycota	0.083	−0.156	0.084	0.198	0.466^*^	0.123	0.071	−0.070
	Rozellomycota	−0.652^*^	0.570^*^	−0.551^*^	−0.619^*^	−0.346	−0.748^*^	−0.700^*^	−0.685^*^
	Unclassified k Fungi	−0.478^*^	0.473^*^	−0.635^*^	−0.378^*^	−0.462^*^	−0.798^*^	−0.304	−0.812^*^
	Olpidiomycota	−0.433^*^	0.550^*^	−0.412^*^	−0.321	−0.359	−0.554^*^	−0.477^*^	−0.625^*^
	Chytridiomycota	0.115	−0.172	−0.423^*^	−0.273	−0.423^*^	−0.234	0.013	−0.394^*^

* indicates *p* < 0.05.

## Discussion

There are differences in the response of plants to acid rain at different pH values (Wang and Zhou, 2010; Yi et al., 2014). When the pH value is reduced to the tolerance range of plants, acid rain damages plant and inhibits their growth (Ju et al., 2017), which is consistent with the results of our study, showing that the plant height and total biomass of *A*. *philoxeroides* and *A*. *sessilis* decreased with the decrease in the pH of acid rain. Under the same simulated acid rain concentration, the damage to *A*. *philoxeroides* was less than that of *A*. *sessilis*, indicating that the invasive plant *A*. *philoxeroides* had stronger acid rain tolerance than the native plant *A*. *sessilis*. The chlorophyll content is greatly affected by acid rain, and the stability of chlorophyll b is stronger when it is damaged by acid rain (Ma et al., 2021), which is consistent with the results of our study. Acid rain inhibited the chlorophyll content of *A*. *philoxeroides* and *A*. *sessilis*. The decrease in chlorophyll content is an important index for judging leaf senescence. The chlorophyll content of *A*. *philoxeroides* under the pH 2.5 treatment was significantly lower than that of CK, indicating that leaf senescence accelerated. However, the leaves of *A*. *sessilis* had begun senescence under the pH 4.5 treatment, which may be due to the morphological structure, cell pH value, chloroplast membrane, or stomatal stability of *A*. *philoxeroides* leaves (Hu et al., 2021). Acid rain caused less damage to the chlorophyll of *A*. *philoxeroides*, thus reducing the degradation of chlorophyll by acid rain. In contrast, chlorophyll b has the function of absorbing and converting light energy, and *A*. *philoxeroides* can maintain the stability of the photosynthetic system to resist adversity damage by regulating chlorophyll b content (Rudiger, 2002). Photosynthesis is the basis of plant production, and the response to acid rain stress is very sensitive (Zhang et al., 2021). Some studies have found that acid rain can inhibit photosynthesis in plants, and the degree of inhibition increases with a decrease in pH (Qiu et al., 2002), which is consistent with the results of our study. The Pn, Gs, and Tr of *A*. *philoxeroides* and *A*. *sessilis* decreased with the decrease in pH value. The Pn of *A*. *philoxeroides* and *A*. *sessilis* decreased significantly under the pH 4.5 treatment, which was consistent with the decreasing trend of Ci. Therefore, the decrease in the photosynthetic rate was due to stomatal factors (Gao et al., 2021). Leaves of *A*. *philoxeroides* and *A*. *sessilis* adapt to acid rain stress by regulating stomata, reducing transpiration, and reducing the entry of acid rain. The invasive plant *A*. *philoxeroides* had a stronger adaptability to acid rain than the native plant *A*. *sessilis*.

A previous study indicated that plant diseases are associated with variations in the composition and structure of the rhizosphere microbial community (Wu et al., 2019, 2021). Rhizosphere microorganisms are the most active life forms in the soil (Heijden et al., 2008). Through diversity analysis of soil microbial community structures, acid rain increased the abundance and diversity of soil microbial communities in *A*. *philoxeroides* and *A*. *sessilis*. This is in contrast to previous studies that found that soil microbial diversity and richness indexes first increased and then decreased with the decrease in pH of acid rain (Wang et al., 2014), which may be the “fertilization effect” caused by short-term acid rain input, and the activation of effective nutrients in soil can resist the negative effects of acid rain and stimulate the reproduction of soil microorganisms (Liu et al., 2020). Soil microbial diversity is the result of the joint influence of many factors, and soil physicochemical properties are the key influencing factors (Kourtev et al., 2002). With the application of acid rain, the soil pH, organic matter, total nitrogen, total phosphorus, total potassium, ammonium nitrogen, and available phosphorus of *A*. *philoxeroides* and *A*. *sessilis* decreased, and these nutrients were significantly negatively correlated with Chao1, ACE, and Shannon indexes of soil bacteria and fungi and significantly positively correlated with the Simpson index, further indicating that acid rain stress led to the loss of soil nutrients to improve the microbial diversity of *A*. *philoxeroides* and *A*. *sessilis*. The higher the microbial diversity, the stronger the stability and stress resistance of the ecosystem (Zhou et al., 2002). In this study, the increase in microbial flora richness and diversity of *A*. *philoxeroides* under acid rain treatment was greater than that of *A*. *sessilis*, indicating that *A*. *philoxeroides* obtained higher soil microbial diversity and formed functional redundancy under acid rain stress. Functional redundancy ensures that the soil stability and resistance of *A*. *philoxeroides* are stronger than that of *A*. *sessilis* under acid rain stress (Chaer et al., 2009).

Different plants can form adaptive microbial communities in the surrounding soil, and the structure of soil microbial communities changes under acid rain stress (Wang et al., 2020). Consistent with the results of this study, there were significant differences in the fungal and bacterial community structures of *A*. *philoxeroides* and *A*. *sessilis* between CK and the simulated acid rain treatment at pH 2.5. High-throughput sequencing showed that the dominant bacterial groups of *A*. *philoxeroides* and *A*. *sessilis* under acid rain treatment were Proteobacteria, Actinobacteriota, Bacteroidota, and Acidobacteriota, and the dominant fungal groups were Ascomycota, Basidiomycota, which was consistent with a previous analysis of microbial communities in *A*. *philoxeroides* and *A*. *sessilis* (Yang et al., 2019). Acid rain did not significantly change the community structure of soil bacterial phyla between *A*. *philoxeroides* and *A*. *sessilis*, and the relative proportion of dominant fungal phyla was significantly different. This may be because the community structure and composition of soil fungi can quickly respond to environmental changes (Wan et al., 2015). Ascomycota tend to be more abundant in stress environments, and their relative abundance increases with the application of nitrogen fertilizer (He et al., 2016). This study also found a significant positive correlation between Ascomycota and total soil nitrogen. The content of soil total nitrogen of *A*. *philoxeroides* and *A*. *sessilis* increased first and then decreased with the decrease in the pH of acid rain, and the relative abundance of Ascomycota also changed accordingly. The decrease in organic matter in soil reduces the richness of available substrate abundance of Basidiomycota and inhibits their growth, which is consistent with the present study. Basidiomycota were positively correlated with organic matter. Ascomycota and Basidiomycota are mostly saprophytic bacteria that are important decomposers in soil (Ma et al., 2013). They can decompose refractory organic matter, such as lignin and keratin, in the soil and promote nutrient circulation and energy flow (Beimforde et al., 2014). Under acid rain stress, the decline in saprophytic microbial communities of *A*. *philoxeroides* was less than that of *A*. *sessilis*, which was consistent with the function of soil bacteria in *A*. *philoxeroides* and *A*. *sessilis*, as it was not affected by acid rain, while the decline of saprophytic vegetative fungi in the soil of *A*. *philoxeroides* was less than that of *A*. *sessilis*.

Previous studies have shown that the root exudates of plants alter the composition and diversity of the rhizosphere microbiome, mediating the abundance of specific pathogens and beneficial microbes in the rhizosphere soil and causing plant diseases (Wu et al., 2016a, 2017, 2019). In this study, fewer plant pathogens were observed in the rhizosphere soil of *A*. *philoxeroides* than in *A*. *sessilis*. This might be because invasive *A*. *philoxeroides* inhibits the growth of pathogens by some specific root exudate. The process and mechanism of this phenomenon are worth further study. The application of acid rain to reduce phosphorus content can lead to the transformation of soil bacterial microbial communities to fungal microbial communities (Lauber et al., 2008). In this study, TP was positively correlated with Acidobacteriota, negatively correlated with Rozellomycota, AP was positively correlated with Bacteroidota, and negatively correlated with Rozellomycota and Olpidiomycota. This is because bacteria have higher specific surface area requirements for phospholipids, and the decrease in soil phosphorus content inhibits bacterial growth (Nottingham et al., 2018). Although acid rain can reduce soil nutrient content, fungi have a strong adaptability to the environment and the ability to obtain nutrients under acidic conditions (DeForest and Scott, 2010).

## Conclusion

In the current study, we provided evidence that simulated acid rain had a significant inhibitory effect on the morphological and physiological traits of invasive *A*. *philoxeroides* and native *A*. *sessilis*, reduced the soil nutrient content, and changed the microbial community structure of the two species. Soil microorganisms of *A*. *philoxeroides* maintained higher diversity by changing species composition, recruiting fewer plant pathogens, and showing better acid rain adaptability and resistance than *A*. *sessilis*. This study provides a new perspective for a better understanding of the plant invasion process.

## Data availability statement

The datasets presented in this study can be found in online repositories. The names of the repository/repositories and accession number(s) can be found at: NCBI—PRJNA863323.

## Author contributions

YLiu and ZZ conceived the study and helped to revise the manuscript. MH, ZH, HC, and YLi collected the samples and analyzed the data. All authors contributed to the article and approved the submitted version.

## Funding

This work was funded by the National Science Foundation of China (grant no. 31772235) and State Key Laboratory of Vegetation and Environmental Change (grant no. LVEC-2022kf01).

## Conflict of interest

The authors declare that the research was conducted in the absence of any commercial or financial relationships that could be construed as a potential conflict of interest.

## Publisher’s note

All claims expressed in this article are solely those of the authors and do not necessarily represent those of their affiliated organizations, or those of the publisher, the editors and the reviewers. Any product that may be evaluated in this article, or claim that may be made by its manufacturer, is not guaranteed or endorsed by the publisher.

## References

[ref1] Averill C. Classen A. (2014). Divergence in plant and microbial allocation strategies explains continental patterns in microbial allocation and biogeochemical fluxes. Ecol. Lett. 17, 1202–1210. doi: 10.1111/ele.12324, PMID: 25040202

[ref2] Beimforde C. Feldberg K. Nylinder S. Rikkinen J. Tuovila H. Drfelt H.. (2014). Estimating the Phanerozoic history of the Ascomycota lineages: combining fossil and molecular data. Mol. Phylogenet. Evol. 78, 386–398. doi: 10.1016/j.ympev.2014.04.024, PMID: 24792086

[ref3] Chaer G. Fernandes M. Myrold D. Bottomley P. (2009). Comparative resistance and resilience of soil microbial communities and enzyme activities in adjacent native forest and agricultural soils. Microb. Ecol. 58, 414–424. doi: 10.1007/s00248-009-9508-x, PMID: 19330551

[ref4] Chen S. Zhou Y. Chen Y. Jia G. (2018). Fastp: an ultra-fast all-in-one FASTQ preprocessor. Bioinformatics 34, i884–i890. doi: 10.1093/bioinformatics/bty560 30423086 PMC6129281

[ref5] Chu H. Y. Sun H. B. Tripathi B. M. Adams J. M. Huang R. Zhang Y. J.. (2016). Bacterial community dissimilarity between the surface and subsurface soils equals horizontal differences over several kilometers in the western Tibetan plateau. Environ. Microbiol. 18, 1523–1533. doi: 10.1111/1462-2920.13236, PMID: 26914676

[ref6] DeForest J. L. Scott L. G. (2010). Available organic soil phosphorus has an important influence on microbial community composition. Soil Sci. Soc. Am. J. 74, 2059–2066. doi: 10.2136/sssaj2009.0426

[ref7] Dolatabadian A. Sanavy S. A. M. M. Gholamhoseini M. Joghan A. K. Majdi M. Kashkooli A. B. (2013). The role of calcium in improving photosynthesis and related physiological and biochemical attributes of spring wheat subjected to simulated acid rain. Physiol. Mol. Biol. Plants 19, 189–198. doi: 10.1007/s12298-013-0165-7, PMID: 24431486 PMC3656184

[ref8] Du E. Z. Dong D. Zeng X. T. Sun Z. Z. Jiang X. F. de Vries W. (2017). Direct effect of acid rain on leaf chlorophyll content of terrestrial plants in China. Sci. Total Environ. 605-606, 764–769. doi: 10.1016/j.scitotenv.2017.06.044, PMID: 28679120

[ref9] Edgar R. C. (2013). UPARSE: highly accurate OTU sequences from microbial amplicon reads. Nat. Methods 10, 996–998. doi: 10.1038/nmeth.2604, PMID: 23955772

[ref10] El-Tarabily K. A. Nassar A. H. Sivasithamparam K. (2007). Promotion of growth of bean (*Phaseolus vulgaris* L.) in a calcareous soil by a phosphate-solubilizing, rhizosphere-competent isolate of Micromonospora endolithica. Appl. Soil Ecol. 39, 161–171. doi: 10.1016/j.apsoil.2007.12.005

[ref11] Falkowski P. G. Fenchel T. Delong E. F. (2008). The microbial engines that drive Earth's biogeochemical cycles. Science 320, 1034–1039. doi: 10.1126/science.1153213, PMID: 18497287

[ref12] Gao Y. F. Rong L. P. Zhao D. H. Zhang J. Q. Chen J. S. (2021). Effects of simulated acid rain on the photosynthetic physiology of *Acer ginnala* seedlings. Can. J. For. Res. 51, 18–24. doi: 10.1139/cjfr-2020-0091

[ref13] Guo W. Li J. M. Hu Z. H. (2012). Effects of clonal integration on growth of *Alternanthera philoxeroides* under simulated acid rain and herbivory. Acta Ecol. Sin. 32, 151–158. doi: 10.5846/stxb201011031575

[ref14] He D. Xiang X. J. He J. S. Wang C. Cao G. M. Adams J.. (2016). Composition of the soil fungal community is more sensitive to phosphorus than nitrogen addition in the alpine meadow on the Qinghai-Tibetan plateau. Biol. Fertil. Soils 52, 1059–1072. doi: 10.1007/s00374-016-1142-4

[ref15] Heijden M. BaRdgett R. D. Straalen N. (2008). The unseen majority: soil microbes as drivers of plant diversity and productivity in terrestrial ecosystems. Ecol. Lett. 11, 296–310. doi: 10.1111/j.1461-0248.2007.01139.x, PMID: 18047587

[ref16] Hu H. Hua W. Shen A. L. Zhou H. K. Sheng L. Lou W. D.. (2021). Photosynthetic rate and chlorophyll fluorescence of barley exposed to simulated acid rain. Environ. Sci. Pollut. Res. 28, 42776–42786. doi: 10.1007/s11356-021-13807-8 33822300

[ref17] Ju S. M. Wang L. P. Yin N. N. Li D. Wang Y. K. Zhang C. Y. (2017). Silicon alleviates simulated acid rain stress of *Oryza sativa* L. seedlings by adjusting physiology activity and mineral nutrients. Protoplasma 254, 2071–2081. doi: 10.1007/s00709-017-1099-7, PMID: 28303353

[ref18] Kourtev P. S. Ehrenfeld J. G. HaGgblom M. (2002). Exotic plant species alter the microbial community structure and function in the soil. Ecology 83, 3152–3166. doi: 10.1890/0012-9658(2002)083[3152:EPSATM]2.0.CO;2

[ref19] Lauber C. Strickland M. S. Bradford M. A. Fierer N. (2008). The influence of soil properties on the structure of bacterial and fungal communities across land-use types. Soil Biol. Biochem. 40, 2407–2415. doi: 10.1016/j.soilbio.2008.05.021

[ref20] Li Y. B. Liang S. W. Du X. F. Kou X. C. Lv X. T. Li Q. (2020). Mowing did not mitigate the negative effects of nitrogen deposition on soil nematode community in a temperate steppe. Soil Ecol. Lett. 3, 1–9. doi: 10.1007/s42832-020-0048-0

[ref21] Liu M. Huang X. Song Y. Tang J. Zhu T. (2019). Ammonia emission control in China would mitigate haze pollution and nitrogen deposition, but worsen acid rain. Proc. Natl. Acad. Sci. 116, 7760–7765. doi: 10.1073/pnas.1814880116, PMID: 30936298 PMC6475379

[ref22] Liu Z. Q. Li D. F. Zhang J. E. Saleem M. Zhang Y. Ma R.. (2020). Effect of simulated acid rain on soil CO_2_, CH_4_ and N_2_O emissions and microbial communities in an agricultural soil. Geoderma 366:114222. doi: 10.1016/j.geoderma.2020.114222

[ref23] Liu X. Zhang B. Zhao W. Wang L. Xie D. Huo W.. (2017). Comparative effects of sulfuric and nitric acid rain on litter decomposition and soil microbial community in subtropical plantation of Yangtze River delta region. Sci. Total Environ. 601-602, 669–678. doi: 10.1016/j.scitotenv.2017.05.151 28577402

[ref24] Liu X. Zhao W. R. Meng M. J. Fu Z. Y. Xu L. H. Zha Y.. (2018). Comparative effects of simulated acid rain of different ratios of SO_4_^2−^ to NO_3_^−^ on fine root in subtropical plantation of China. Sci. Total Environ. 618, 336–346. doi: 10.1016/j.scitotenv.2017.11.073, PMID: 29132001

[ref25] Lv Y. N. Wang C. Y. Jia Y. Y. Wang W. W. Ma X. Du J. J.. (2014). Effects of sulfuric, nitric, and mixed acid rain on litter decomposition, soil microbial biomass, and enzyme activities in subtropical forests of China. Appl. Soil Ecol. 79, 1–9. doi: 10.1016/j.apsoil.2013.12.002

[ref26] Ma S. L. Liu X. Jia Z. H. Meng M. J. Li C. Ren Q.. (2021). Response of *Quercus acutissima* foliage to different types of simulated acid rain. Atmos. Pollut. Res. 12:101112. doi: 10.1016/j.apr.2021.101112

[ref27] Ma A. Z. Zhuang X. L. Wu J. Mei C. M. M. Lv D. Liu C. Z.. (2013). Ascomycota members dominate fungal communities during straw residue decomposition in arable soil. PLoS One 8:e66146. doi: 10.1371/journal.pone.0066146, PMID: 23840414 PMC3688710

[ref28] Mago T. Salzberg S. L. (2011). FLASH: fast length adjustment of short reads to improve genome assemblies. Bioinformatics 27, 2957–2963. doi: 10.1093/bioinformatics/btr507, PMID: 21903629 PMC3198573

[ref29] Meng F. B. Yang X. D. Duan L. C. Naidu R. Nuruzzaman M. Semple K. T. (2019). Influence of pH, electrical conductivity and ageing on the extractability of benzo[a]pyrene in two contrasting soils. Sci. Total Environ. 690, 647–653. doi: 10.1016/j.scitotenv.2019.06.445, PMID: 31301505

[ref30] Mohsen J. Elaheh N. (2012). The impact of acid rain on phosphorus leaching from a sandy loam calcareous soil of western Iran. Environ. Earth Sci. 66, 311–317. doi: 10.1007/s12665-011-1240-4

[ref31] Nottingham A. T. Hicks L. C. Ccahuana A. J. Q. Salinas N. Baath E. Meir P. (2018). Nutrient limitations to bacterial and fungal growth during cellulose decomposition in tropical forest soils. Biol. Fertil. Soils 54, 219–228. doi: 10.1007/s00374-017-1247-4

[ref32] Pan X. Y. Geng Y. P. Zhang W. J. Li B. Chen J. K. (2006). Cover shift and morphological plasticity of invasive *Alternanthera philoxeroides* along ariparian zone in South China. J. Plant Ecol. 30, 835–843. doi: 10.17521/cjpe.2006.0106

[ref33] Qiu D. L. Liu X. H. Guo S. Z. (2002). Effects of simulated acid rain and chlorophyll a fluorescence parameters in leaves of longan. Acta Phytoecol. Sin. 26, 441–446. doi: 10.1006/jfls.2001.0409

[ref34] Ramlall C. Varghese B. Ramdhani S. Pammenter N. W. Bhatt A. Berjak P.. (2015). Effects of simulated acid rain on germination, seedling growth and oxidative metabolism of recalcitrant-seeded *Trichilia dregeana* grown in its natural seed bank. Physiol. Plant. 153, 149–160. doi: 10.1111/ppl.12230, PMID: 24835442

[ref35] Ren J. H. Liu X. L. Yang W. P. Yang X. X. Li W. G. Xia Q.. (2021). Rhizosphere soil properties, microbial community, and enzyme activities: short-term responses to partial substitution of chemical fertilizer with organic manure. J. Environ. Manage. 299:113650. doi: 10.1016/j.jenvman.2021.113650, PMID: 34481370

[ref36] Rudiger W. (2002). Biosynthesis of chlorophyll b and the chlorophyll cycle. Photosynth. Res. 74, 187–193. doi: 10.1023/A:1020959610952 16228557

[ref37] Shafer S. R. (1992). Responses of microbial populations in the rhizosphere to deposition of simulated acidic rain onto foliage and/or soil. Environ. Pollut. 76, 267–278. doi: 10.1016/0269-7491(92)90146-2 15091992

[ref38] Shu X. Zhang K. R. Zhang Q. F. Wang W. B. (2019). Ecophysiological responses of *Jatropha curcas* L. seedlings to simulated acid rain under different soil types. Ecotoxicol. Environ. Saf. 185:109705. doi: 10.1016/j.ecoenv.2019.109705, PMID: 31561080

[ref39] Shu X. Zhang K. R. Zhang Q. F. Wang W. B. (2021). Tolerant mechanism of *Jatropha curcas* L. roots to acid rain in soils with different acid-buffering capacities. Acta Physiol. Plant. 43, 1–11. doi: 10.1007/s11738-021-03329-8

[ref40] Stakenrandt E. Goebel B. (1994). Taxonomic note: a place for DNA-DNA reassociation and 16S rRNA sequence analysis in the present species definition in bacteriology. Int. J. Syst. Bacteriol. 44, 846–849.

[ref41] Sun Y. Ding J. Q. Frye M. (2010). Effects of resource availability on tolerance of herbivory in the invasive *Alternanthera philoxeroides* and the native *Alternanthera sessilis*. Weed Res. 50, 527–536. doi: 10.1111/j.1365-3180.2010.00822.x

[ref42] Wan X. H. Huang Z. Q. He Z. M. Yu Z. P. Wang M. H. Davis M. R.. (2015). Soil C:N ratio is the major determinant of soil microbial community structure in subtropical coniferous and broadleaf forest plantations. Plant and Soil 387, 103–116. doi: 10.1007/s11104-014-2277-4

[ref43] Wang L. Chen Z. Shang H. Wang J. Zhang P. Y. (2014). Impact of simulated acid rain on soil microbial community function in Masson pine seedlings. Electron. J. Biotechnol. 17, 199–203. doi: 10.1016/j.ejbt.2014.07.008

[ref44] Wang L. C. Liu Y. Zhu X. M. Zhang Z. Huang X. Q. (2021a). Identify potential allelochemicals from *Humulus scandens* (Lour.) Merr. Root extracts that induce allelopathy on *Alternanthera philoxeroides* (Mart.) Griseb. Sci. Rep. 11, 7068–7076. doi: 10.1038/s41598-021-86656-7, PMID: 33782496 PMC8007610

[ref45] Wang N. Pan X. C. Bai S. B. Zhnag T. (2020). Effects of acid rain on root morphology and distribution pattern in the buffer zone of broad-leaved forest invaded by Moso bamboo. Acta Ecol. Sin. 40, 4670–4678. doi: 10.5846/stxb201909031826

[ref46] Wang Y. Xiong Y. T. Wang Y. Li Q. J. (2021b). Long period exposure to serious cadmium pollution benefits an invasive plant (*Alternanthera philoxeroides*) competing with its native congener (*Alternanthera sessilis*). Sci. Total Environ. 786:147456. doi: 10.1016/j.scitotenv.2021.147456, PMID: 33965821

[ref47] Wang K. Yang J. Chen J. K. (2009). The applications of congeneric comparisons in plant invasion ecology. Biodivers. Sci. 17, 353–361. doi: 10.3724/SP.J.1003.2009.09055

[ref48] Wang L. H. Zhou Q. (2010). Responses of rice seed germination to acid rain stress. Seed Sci. Technol. 38, 26–35. doi: 10.15258/sst.2010.38.1.03

[ref49] Wei H. Liu W. Zhang J. E. Qin Z. (2017). Effects of simulated acid rain on soil fauna community composition and their ecological niches. Environ. Pollut. 220, 460–468. doi: 10.1016/j.envpol.2016.09.088, PMID: 27697382

[ref50] Wei H. Ma R. Zhang J. E. Zhou L. Y. Liu Z. Q. Fan Z. Y.. (2020). Quality dependence of litter decomposition and its carbon, nitrogen and phosphorus release under simulated acid rain treatments. Environ. Sci. Pollut. Res. 27, 19858–19868. doi: 10.1007/s11356-020-08423-x, PMID: 32227303

[ref51] Whitaker J. Ostle N. Nottingham A. T. Ccahuana A. Salinas N. Bardgett R. D.. (2014). Microbial community composition explains soil respiration responses to changing carbon inputs along an Andes-to-Amazon elevation gradient. J. Ecol. 102, 1058–1071. doi: 10.1111/1365-2745.12247, PMID: 25520527 PMC4263258

[ref52] Wu J. P. Liang G. H. Hui D. F. Deng Q. Xiong X. Qiu Q. Y.. (2016b). Prolonged acid rain facilitates soil organic carbon accumulation in a mature forest in southern China. Sci. Total Environ. 544, 94–102. doi: 10.1016/j.scitotenv.2015.11.025, PMID: 26657252

[ref53] Wu G. Ling J. Xu Y. P. Zhao D. Q. Liu Z. X. Wen Y.. (2022). Effects of soil warming and straw return on soil organic matter and greenhouse gas fluxes in winter wheat seasons in the North China plain. J. Clean. Prod. 356:131810. doi: 10.1016/j.jclepro.2022.131810

[ref54] Wu H. M. Qin X. J. Wang J. Y. Wu L. K. Chen J. Fan J. K.. (2019). Rhizosphere responses to environmental conditions in *Radix pseudostellariae* under continuous monoculture regimes. Agric. Ecosyst. Environ. 270, 19–31. doi: 10.1016/j.agee.2018.10.014

[ref55] Wu H. M. Wu H. M. Qin X. J. Lin M. H. Zhao Y. L. Rensing C.. (2021). Replanting disease alters the faunal community composition and diversity in the rhizosphere soil of *radix pseudostellariae*. Agric. Ecosyst. Environ. 310:107304. doi: 10.1016/j.agee.2021.107304

[ref56] Wu H. M. Wu L. K. Wang J. Y. Zhu Q. Sheng L. Xu J. H.. (2016a). Mixed phenolic acids mediated proliferation of pathogens *Talaromyces helicus* and *Kosakonia sacchari* in continuously monocultured *radix pseudostellariae* rhizosphere soil. Front. Microbiol. 7:335. doi: 10.3389/fmicb.2016.00335 27014250 PMC4795122

[ref57] Wu H. M. Wu L. K. Zhu Q. Wang J. Y. Qin X. J. Xu J. H.. (2017). The role of organic acids on microbial deterioration in the *radix pseudostellariae* rhizosphere under continuous monoculture regimes. Sci. Rep. 7:3497. doi: 10.1038/s41598-017-03793-8, PMID: 28615734 PMC5471291

[ref58] Xu H. Q. Zhang J. E. OuYang Y. Lin L. Quan G. M. Zhao B. L.. (2015). Effects of simulated acid rain on microbial characteristics in a lateritic red soil. Environ. Sci. Pollut. Res. 22, 18260–18266. doi: 10.1007/s11356-015-5066-6, PMID: 26201661

[ref59] Yang B. F. Zhang X. Zagorchev L. Li J. M. Frey B. Li M. H. (2019). Parasitism changes rhizospheric soil microbial communities of invasive *Alternanthera philoxeroides*, benefitting the growth of neighboring plants. Appl. Soil Ecol. 143, 1–9. doi: 10.1016/j.apsoil.2019.05.025

[ref60] Yi L. T. Liu M. H. Yu S. Q. Yu F. Yin X. M. (2014). Effects of simulated acid rain stress on chlorophyll fluorescence characteristics and growth in leaves of *Lithocarpus glaber* and *Schima superba* seedlings. Asian J. Chem. 26, 4619–4622. doi: 10.14233/ajchem.2014.16141

[ref61] Zhang Y. Y. Tian C. Yu T. Dayananda B. Fu B. Senaratne S. L.. (2021). Differential effects of acid rain on photosynthetic performance and pigment composition of the critically endangered *Acer amplum* subsp. *catalpifolium*. Glob. Ecol. Conserv. 30:e01773. doi: 10.1016/j.gecco.2021.e01773

[ref62] Zhang C. Y. Yi X. Q. Gao X. Z. Wang M. H. Shao C. Y. Lv Z. D.. (2020). Physiological and biochemical responses of tea seedlings (*Camellia sinensis*) to simulated acid rain conditions. Ecotoxicol. Environ. Saf. 192:110315. doi: 10.1016/j.ecoenv.2020.110315, PMID: 32058162

[ref63] Zheng Y. F. Wang Y. Q. Zheng Y. L. Li Y. F. (2022). Effects of simulated acid rain on soil enzyme activity and related chemical indexes in woodlands. Forests 13:860. doi: 10.3390/f13060860

[ref64] Zhou J. Z. Xia B. C. Treves D. S. Wu L. Y. Marsh T. L. O’Neill R. V.. (2002). Spatial and resource factors influencing high microbial diversity in soil. Appl. Environ. Microbiol. 68, 326–334. doi: 10.1128/AEM.68.1.326-334.2002, PMID: 11772642 PMC126564

